# Pendant Ancillary Ligand Switches Off Auto-Oxidation of Group 13 Metal Alkyl Compounds Bearing Non-Bulky Alkyl Groups

**DOI:** 10.1371/journal.pone.0100626

**Published:** 2014-06-19

**Authors:** Issam Kobrsi

**Affiliations:** Department of Chemistry, the Petroleum Institute, Abu Dhabi, United Arab Emirates; Reader in Inorganic Chemistry, United Kingdom

## Abstract

The reaction of 3,5-di-2-pyridyl-1,2,4-triazole with excess Al(CH_3_)_3_ and Ga(CH_3_)_3_ afforded (3,5-di-2-pyridyl-1,2,4-triazolate)Al(CH_3_)_2_•3Al(CH_3_)_3_ (**1**) and (3,5-di-2-pyridyl-1,2,4-triazolate) Ga(CH_3_)_2_•3Ga(CH_3_)_3_ (**2**) respectively. **1** and **2** reacted with oxygen gas to produce (CH_3_)_2_M(µ-3,5-di-2-pyridyl-1,2,4-triazolate)(µ-OCH_3_)M(CH_3_)_2_ (M = Al, **3**; M = Ga, **4**). **3** and **4** contain the non-bulky dimethylalumino moiety, yet they are indefinitely stable in the presence of oxygen gas. This increased stability towards oxygen is due to ancillary 2-pyridyl groups bonding to the metal centers producing a pseudo-trigonal pyramidal Al and Ga environments. This environment blocks oxygen from further inserting into the M–C bond. The Al–N(pyridine) and Ga–N(pyridine) bonds reported herein are extremely elongated yet inactive towards dissociation due to the chelate effect.

## Introduction

The atmospheric oxidation of group 13 metal alkyls is usually very rapid and uncontrollable, often resulting in combustion. [1]–[3] However, controlled oxidation by limited exposure to oxygen gas results in the production of organoperoxide compounds, or alkoxide compounds through the decomposition of the former. [3]–[7] It has been well established that the formation of the alkoxide occurs through an intermolecular oxygen transfer, not an intramolecular one. [1] Thus, it is possible to isolate stable complexes bearing a strongly reducing metal-alkyl bond in close proximity of a strongly oxidizing metal-peroxide bond. [1], [3]–[5] However, it was demonstrated that spatial requirement plays an important part in the oxidation of group 13 metal alkyls. [7] Compounds of the general formula [R_2_AlL]_2_, where L = pyrazolate, readily react with O_2_ to produce an alkoxide when the alkyl group R is non-bulky, such as R = Me, Et; whereas they are stable towards reaction with O_2_ when R is bulky, such as R = *^t^*Bu. There are no examples of a non-bulky alkyl group bonded to a tetrahedral group 13 metal that can resist oxidation to alkoxide or peroxide upon exposure to oxygen gas. I have previously synthesized a set of aluminum and gallium compounds bearing methyl groups of the type (3,5-di-2-pyridyl-1,2,4-triazolate)M(CH_3_)_2_•nM(CH_3_)_3_ (M = Al, n = 3, **1**; M = Ga, n = 1, **2**) in an unpublished work. [8] These complexes readily reacted with air to produce (CH_3_)_2_M(µ-3,5-di-2-pyridyl-1,2,4-triazolate)(µ-OCH_3_)M(CH_3_)_2_ (M = Al, **3**; M = Ga, **4**). **3** and **4** contain a non-bulky dimethylalumino moiety, yet they are indefinitely stable in the presence of oxygen gas. No further scrutiny was given to these complexes or reactions. Recently I became interested in pursuing complexes that bear resemblance to methylalumoxane (MAO), a common co-catalyst. This rekindled my interest in the above compounds and I set out to understand the reaction further by elucidating its mechanism. I hereby report the mechanism and the insight this reaction brings into the corrosion protection of aluminum.

## Results

### Synthetic Aspects

**Figure 1:**
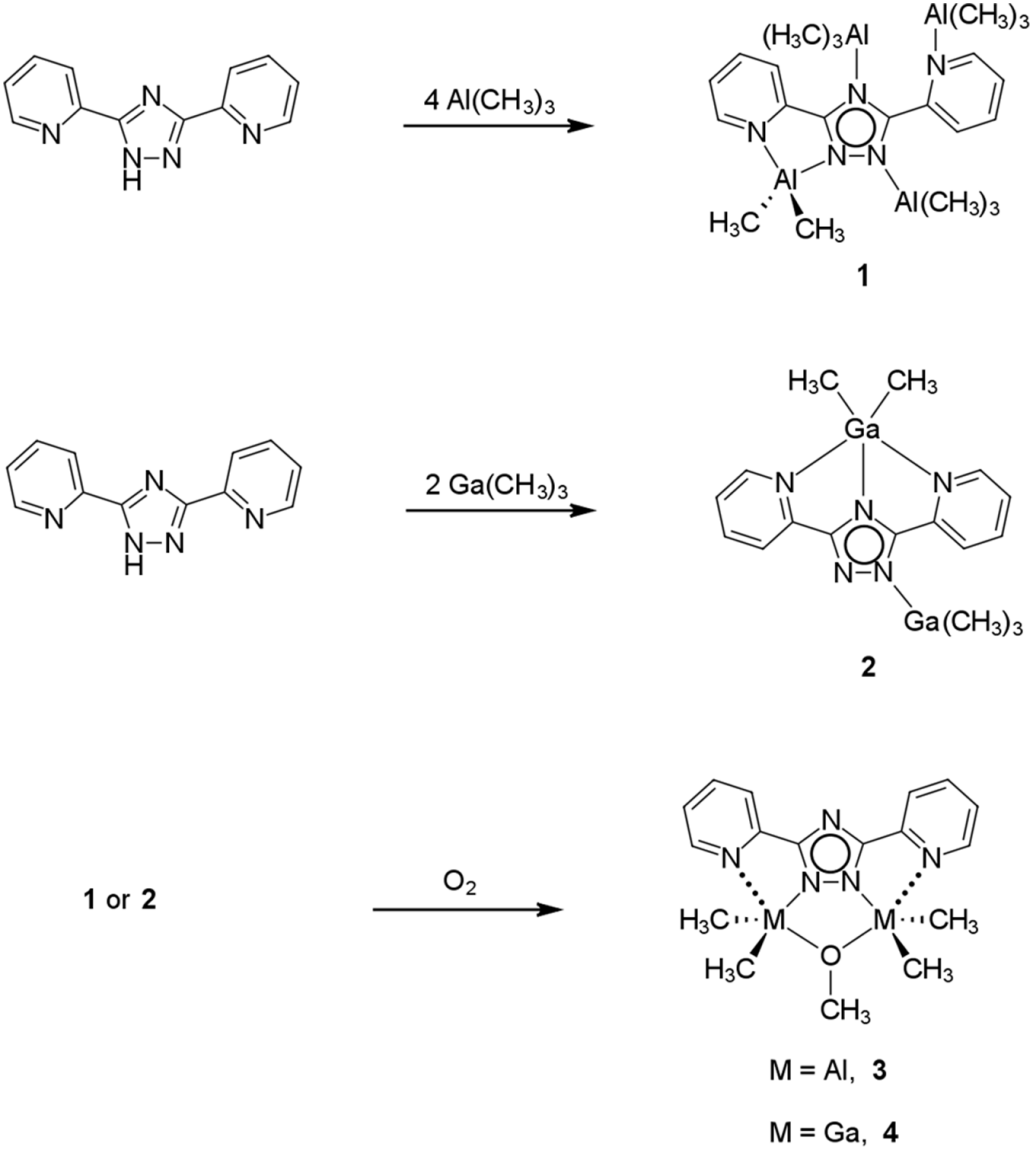
Synthesis scheme of 1–4.

**1** and **2** were synthesized by reacting 3,5-di-2-pyridyl-1,2,4-triazole with excess Al(CH_3_)_3_ and Ga(CH_3_)_3_ in toluene under an atmosphere of argon (Figure 1). **3** and **4** comprise an oxidation resistant [M(CH_3_)_2_]^+^ moiety that is protected by pendant pyridyl groups resulting in a pseudo-trigonal bipyramidal geometry around M with the longest M–N(pyridine) reported bond distances to date. **1**–**4** have been characterized by spectral analysis and X-ray measurements.

When a toluene solution of **1** or **2** was exposed to air undisturbed at room temperature, **3** and **4** precipitated as colorless crystals in high yields. The reaction was fairly quick and crystal formation was observed within 5 minutes. **3** and **4** were indefinitely stable in the presence of air at room temperature.

### X-ray Crystal Structures

**Figure 2:**
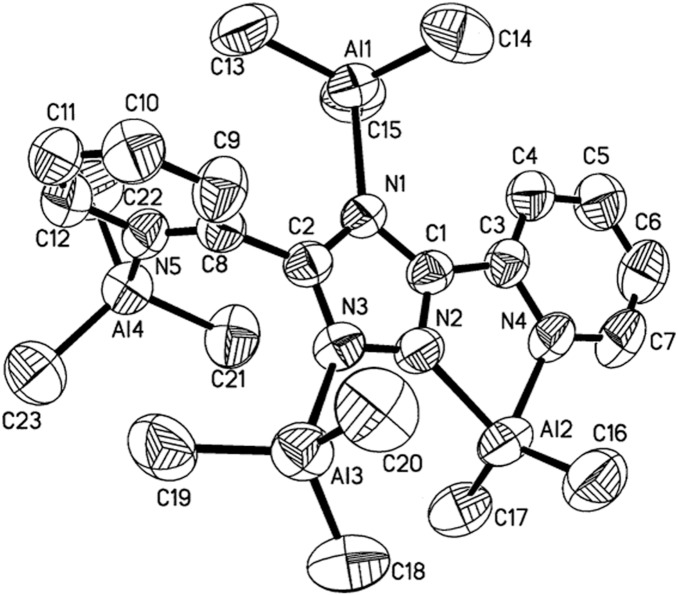
Molecular structure of 1 (50% probability thermal ellipsoids).

**Figure 3:**
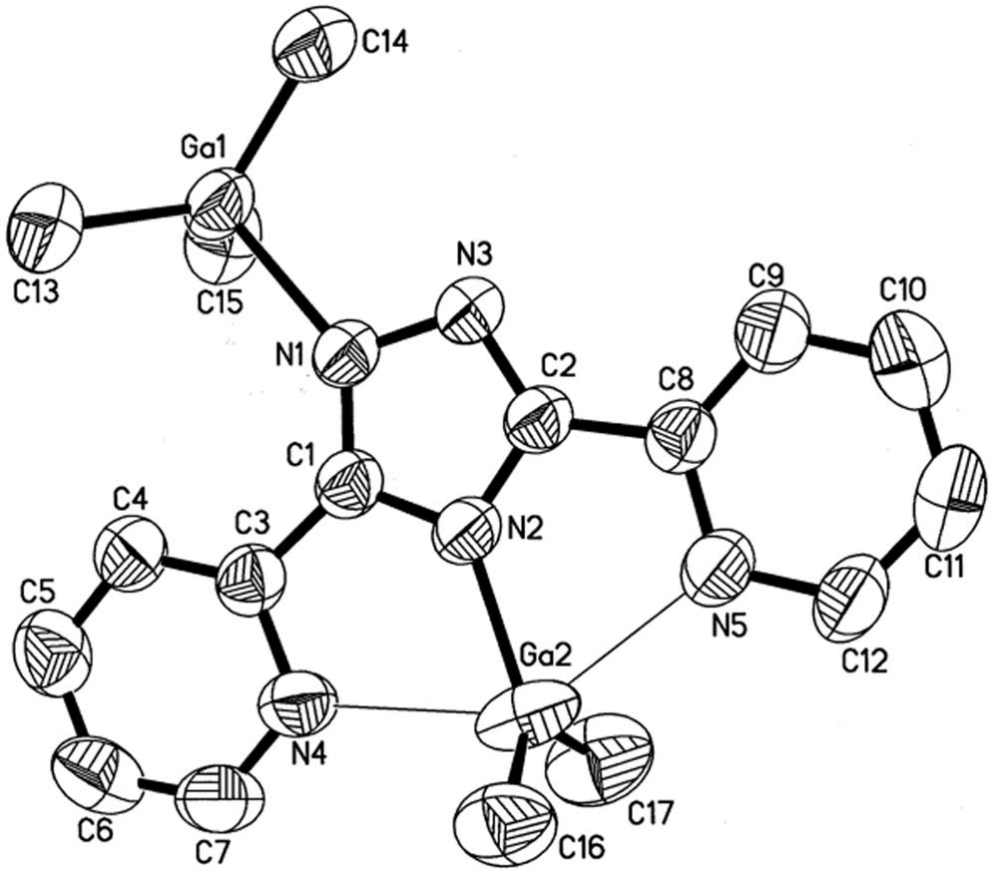
Molecular structure of 2 (50% probability thermal ellipsoids).

**Figure 4:**
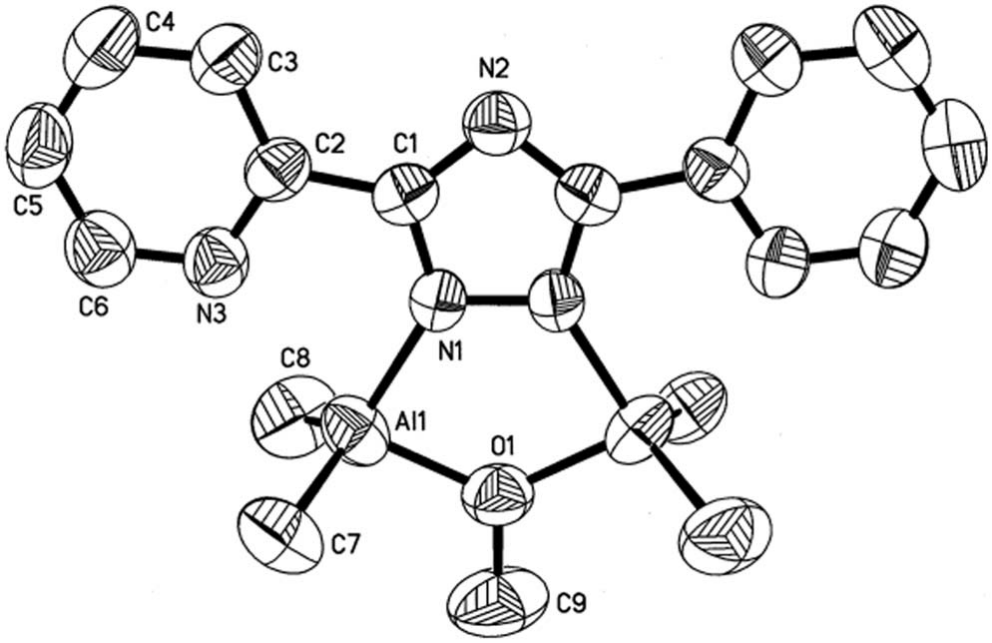
Molecular structure of 3 (50% probability thermal ellipsoids).

**Figure 5:**
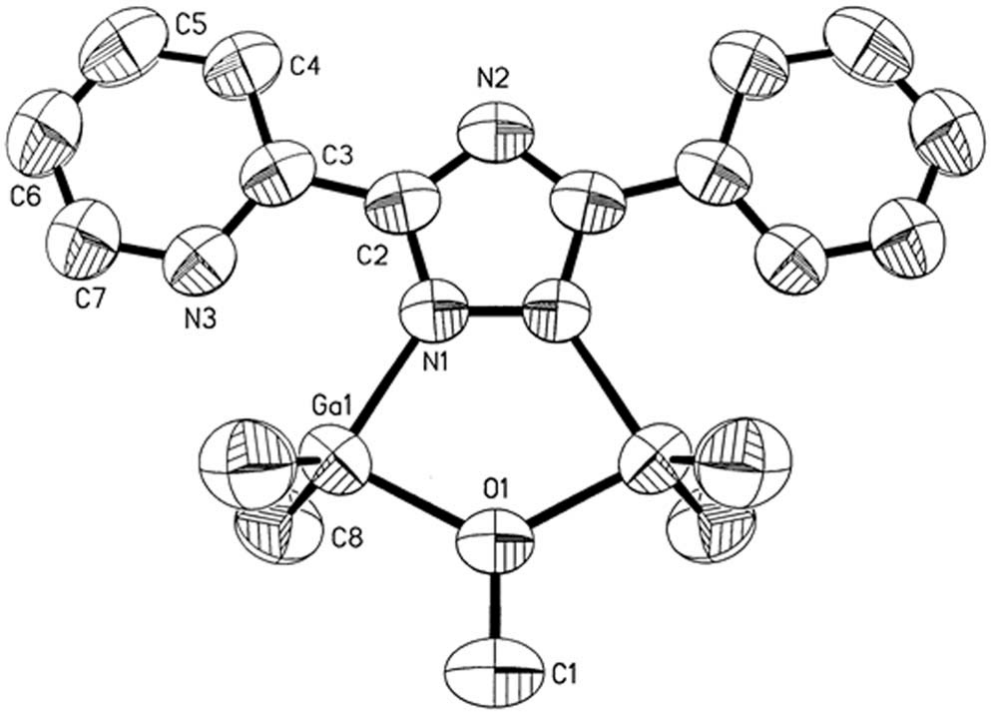
Molecular structure of 4 (50% probability thermal ellipsoids).

The X-ray crystal structures of **1**–**4** were obtained to establish the solid state configurations. Experimental crystallographic data are summarized in Table 1 and selected bond lengths and angles are presented in Tables 2–5. Representative perspective views of **1**–**4** are shown in Figures 2–5. The molecular structure of **1** displays a [(CH_3_)_2_Al]^+^ moiety bridging N^1^ and N(pyridine). All other N atoms are bonded to a trimethylaluminum moiety. The bond distances and angles are as expected. [9], [10] The molecular structure of **2** displays a distorted trigonal bipyramidal environment around the gallium with the equatorial angles ranging from 112.4(2)° to 131.5(2)°. The axial N(4)–Ga(2)–N(5) angle is distorted from linear to 141.54°. The distances from Ga to N(pyridine) are fairly long ranging from 2.458(3) Å to 2.465(3) Å. The coordination of the [Ga(CH_3_)_2_]^+^ moiety is a novel κ^3^-N^4^, N’, N”. The complex also displays a Ga(CH_3_)_3_ adduct to N^1^ with no unusual bonding. The molecular structures of **3** and **4** show very similar features. The triazolato cores, pyridyl groups, metal atoms, and methoxy groups all lie within the same plane. The triazolato ligand bridges two metal atoms in a κ^2^-N^1^, N’ fashion. Most notable are the extremely long Al–N(pyridine) and Ga–N(pyridine) bonds at 2.614 Å and 2.774 Å respectively.

**Table 1:** Experimental Crystallographic data for **1**–**4**.

	1	2	3	4
Empirical formula	C_26_H_48_Al_4_N_5_	C_20.5_H_27_Ga_2_N_5_	C_17_H_23_Al_2_N_5_O	C_17_H_23_Ga_2_N_5_O
Fw	538.61	482.41	367.36	452.84
Space group	P2_1_/n	P-1	C2/c	C2/c
a (Å)	11.3716(16)	9.4967(16)	20.462(2)	20.537(6)
b (Å)	19.732(2)	10.3550	13.5991(14)	13.319(4)
c (Å)	16.223(2)	13.279(2)	7.4024(6)	7.549(2)
β (deg)	110.214(1)	75.471(4)	99.089(2)	90
V (Å^3^)	3416.0(8)	1147.8(3)	2033.9(3)	2064.9(10)
Z	4	2	4	4
Λ	0.71073	0.71073	0.71073	0.71073
Calcd (g cm^−3^)	1.047	1.396	1.200	1.457
µ (mm^−1^)	0.157	2.361	0.157	2.623
R(F)^a^ (%)	5.17	4.95	4.61	2.84
Rw(F)^b^ (%)	13.29	12.74	11.83	6.58

**Table 2:** Selected bond lengths (Å) and angles (deg) for **1**.

Al(2)–N(2)	1.963(2)	Al(2)–N(4)	1.993(2)
Al(3)–N(3)	2.063(2)	Al(1)–N(1)	2.079(2)
Al(4)–N(5)	2.077(2)	Al(2)–C(16)	1.931(3)
Al(2)–C(17)	1.942(3)	N(2)–C(1)	1.324(3)
C(16)–Al(2)–N(4)	106.67(13)	C(16)–Al(2)–N(2)	114.63(13)
C(16)–Al(2)–C(17)	127.29(15)	N(2)–Al(2)–N(4)	79.85(10)

**Table 3:** Selected bond lengths (Å) and angles (deg) for **2**.

Ga(1)–N(1)	2.124(3)	Ga(2)–N(2)	1.938(3)
Ga(2)–N(4)	2.465(3)	Ga(2)–N(5)	2.458(3)
Ga(1)–C(13)	1.988(5)	Ga(2)–C(16)	1.929(4)
N(2)–C(1)	1.332(5)	N(1)–N(3)	1.383(4)
C(16)–Ga(2)–C(17)	131.5(2)	N(2)–Ga(2)–C(16)	116.03(18)
C(16)–Ga(2)–N(5)	98.03(17)	N(5)–Ga(2)–N(4)	141.54

**Table 4:** Selected bond lengths (Å) and angles (deg) for **3**.

Al(1)–O(1)	1.8787(13)	Al(1)–N(1)	1.9223(19)
Al(1)–C(7)	1.951(3)	Al(1)–C(8)	1.951(4)
N(1)–C(1)	1.332(3)	N(2)–C(1)	1.329(3)
O(1)–C(9)	1.439(4)	N(3)–Al(1)	2.614
O(1)–Al(1)–N(1)	85.27(8)	O(1)–Al(1)–C(7)	103.78(16)
N(1)–Al(1)–C(7)	114.84(13)	O(1)–Al(1)–N(3)	152.68

**Table 5:** Selected bond lengths (Å) and angles (deg) for **4**.

Ga(1)–O(1)	1.9652(12)	Ga(1)–C(9)	1.934(10)
Ga(1)–C(8)	1.962(7)	Ga(1)–N(1)	2.005(2)
O(1)–C(1)	1.431(7)	N(1)–C(2)	1.333(3)
C(2)–N(2)	1.337(4)	N(3)–Ga(1)	2.774
C(9)–Ga(1)–O(1)	106.6(6)	C(9)–Ga(1)–C(8)	127.59(18)
O(1)–Ga(1)–C(8)	102.5(6)	O(1)–Ga(1)–N(3)	151.78

## Discussion

The reaction between **1** and air was studied to establish if the reactant was H_2_O or O_2_. When **1** was reacted directly with H_2_O, a white insoluble solid precipitated. This solid isolated, washed with toluene and dried. It did not dissolve in any NMR solvent and its melting point was very high and could not be established using conventional melting point apparatus. It was thus assumed to be aluminum oxide and it was discarded. This made it clear that the source of oxygen in complexes **3** and **4** was not H_2_O.

The reaction between **1** and dry oxygen was also studied. The use of scrubbers was deemed essential to keep moisture out due to the relatively high environmental humidity. The reaction with dry oxygen produced **3** in similar fashion to the reaction with air, thus showing that the source of the oxygen atoms in **3** and 4 is indeed oxygen gas and not water.

**Figure 6:**
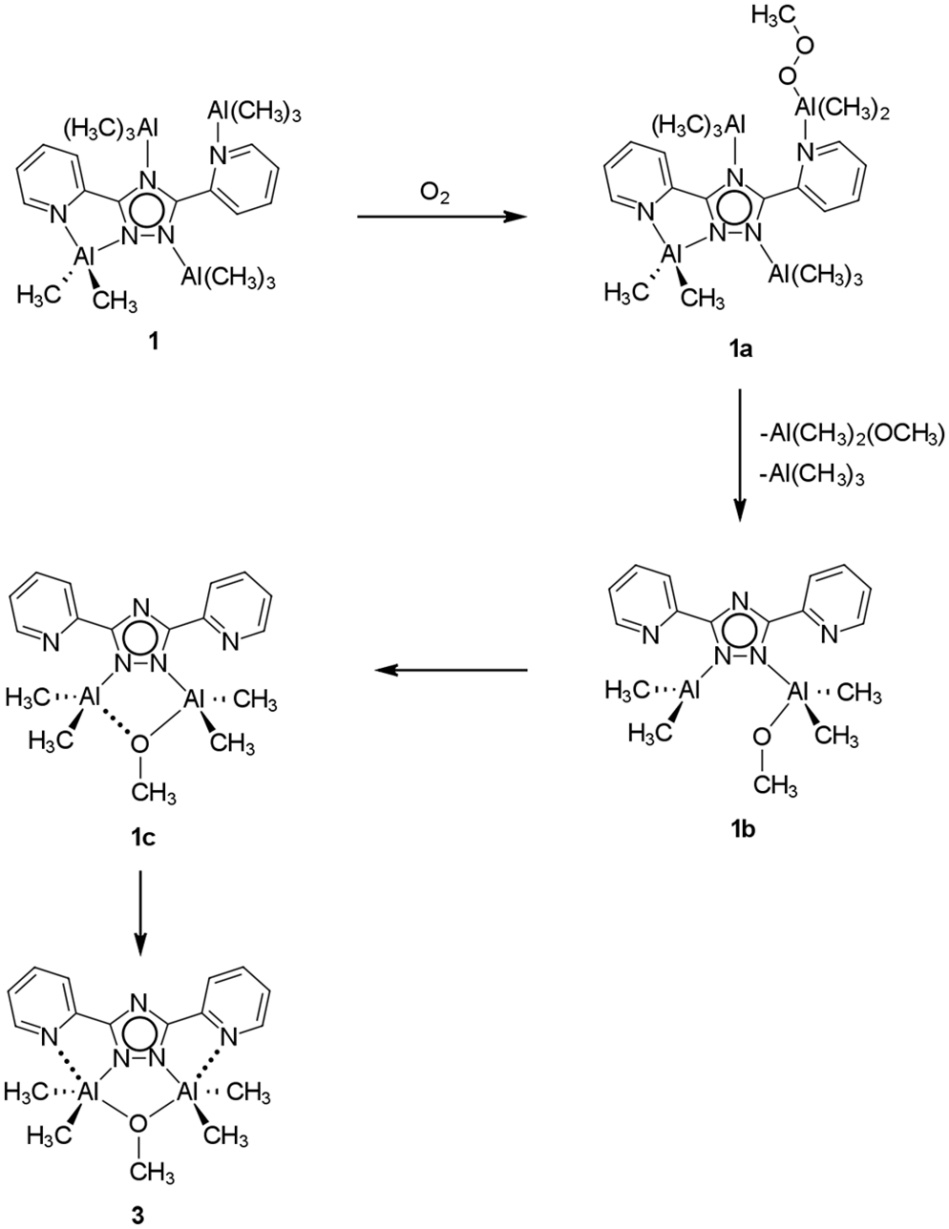
Proposed mechanism for the insertion of O_2_ into 1.

The reaction between **1** and O_2_ can be broken down into steps leading to the protection of the [M(CH_3_)_2_]^+^ moieties from further reaction with oxygen as illustrated in Figure 6. In the first step, oxygen gas is inserted into a M–C bond of the M(CH_3_)_3_ adducts to form the corresponding peroxide **1a**. [3]–[7] In the second step, an oxygen transfer occurs between the peroxide and another adduct within **1a** to form the methoxide **1b**. [1] In the third step, the methoxide group bridges the nearby [M(CH_3_)_2_]^+^ moiety forming a tetrahedral environment in **1c** that is susceptible to reaction with oxygen gas. In the fourth step of this reaction an intramolecular rearrangement occurs by which the susceptible tetrahedral centers of the methoxy-bridged metal centers bind with the pyridyl groups thus preventing any further reaction with oxygen. There is no evidence whether steps 3 and 4 occur sequentially or simultaneously.

**Figure 7:**
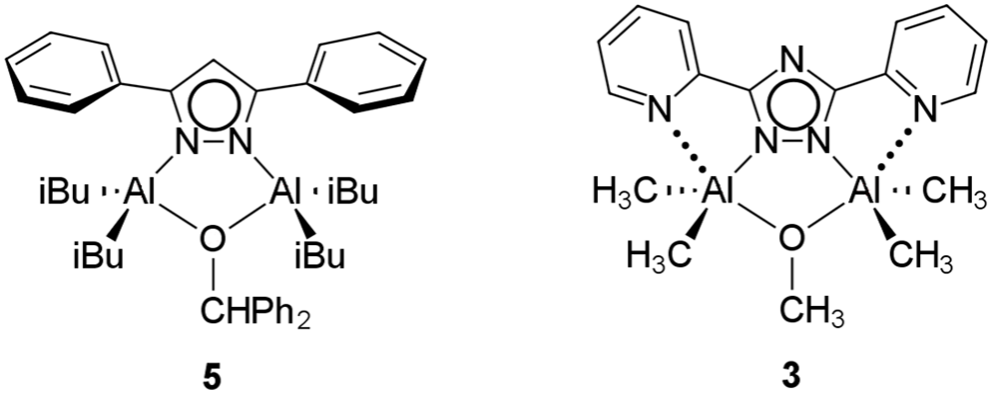
A direct comparison between the structures of oxygen-sensitive 5 and oxygen-stable 3 emphasizing the presence of the Al–N(pyridine) bonds in 3 that contribute to the oxygen-stability.

Direct comparison can be made between **3** and **4**, and the similar compound (^i^Bu)_2_Al(µ-3,5-diphenylpyrazolate)(µ-OCHPh_2_)Al(^i^Bu)_2_ (**5**) (Figure 7). [11] **5** is extremely air sensitive. The tetrahedral aluminum center in **5** is susceptible to attack by O_2_.

The resulting bond between Al/Ga and N(pyridine) is unusually long. For **3**, the Al–N distance is 2.614 Å. The longest reported Al–N(pyridine) distances are 2.262 [12] and 2.254 Å [13], which is 13% to 14% shorter than **3**. For **4**, the Ga–N distance is 2.774 Å. The longest reported Ga–N(pyridine) distance is 2.434 Å [14], which is 12% shorter than **4**. The lengths of these bonds indicate that they should be weak and replaceable by other electron donors such as oxygen gas. However, the length of the bond is offset by the chelate effect making the bonds less likely to dissociate. It has been suggested that the reaction of group 13 metal alkyls with oxygen gas initially go through a five-coordinate intermediate followed by insertion of O_2_ into a M–C bond to generate an Al–OOR moiety. [7] It is for this reason that compounds **3** and **4** are stable towards the reaction with oxygen, due to the pyridyl groups having negligible lability and thus blocking the coordination of the O_2_ by blocking the formation of the metastable five-coordinate peroxo intermediate.

This mechanism provides insight into how these pyridyl triazole ligands can also act as corrosion inhibitors for aluminum, and further studies are underway to determine their feasibility. Further studies are also being performed to establish conditions under which the M–N(pyridine) bond in **3** and **4** can be broken.

## Conclusions

In summary, It has been demonstrated how a bond between aluminum or gallium and a non-bulky alkyl group can be protected against reaction with oxygen using a pendant ancillary ligand. The M[(CH_3_)_2_]^+^ moiety in complexes **1** and **2** remained intact upon reaction with oxygen, and the resulting methoxy-bridged complexes **3** and **4** were protected against further reaction with oxygen by the coordination of the pyridyl group of the triazolate ligand. These complexes exhibit features similar to the theorized MAO structure, and their evaluation as cocatalysts is also underway.

## Experimental Section

### General Considerations

All reactions other than ligand syntheses were performed under an inert atmosphere of argon using either glovebox or Schlenk line techniques. Toluene and hexane were distilled from sodium and phosphorus pentoxide respectively. Trimethylaluminum and trimethylgallium were purchased from Strem Chemicals and used as received.

^1^H and ^13^C NMR were obtained at 500 MHz and 125 MHz, respectively, in benzene-*d_6_*. Infrared spectra were obtained using Nujol as the medium. Elemental analyses were performed by Midwest Microlab, Indianapolis, IN. Melting points were obtained on a Haake-Buchler HBI digital melting point apparatus and were uncorrected.

#### Preparation of tris(trimethylaluminum)(3,5-di-2-pyridyl-1,2,4-triazolato)dimethylaluminum (1)

A 100 mL Schlenk flask equipped with a stir bar and a rubber septum was charged with 3,5-di-2-pyridyl-1,2,4-triazole (0.300 g, 1.340 mmol). Toluene (50 mL) was added and the resulting white suspension was stirred. Trimethylaluminum (0.390 g, 5.380 mmol) was introduced and immediate evolution of methane was observed. The resulting pale yellow solution was allowed to stir for 4 hours after which the volume of toluene was reduced to approximately 20 mL and the flask was stored undisturbed at –20°C. Complex **1** (0.160 g, 24%) was isolated as extremely air sensitive pale yellow cubic crystals. Mp 62°C dec.; ^1^H NMR (C_6_D_6_, 18°C, δ) 8.08 (m, 2H, CH–C*H*–N), 7.81 (m, 2H, aryl C*H*), 6.96 (m, 2H, aryl C*H*), 6.49 (m, 2H, aryl C*H*), –0.125 (s, 27H, Al(C*H*_3_)_3_), –0.516 (s, 6H, Al(C*H*_3_)_2_); ^13^C NMR (C_6_D_6_, 18°C, δ) 161.4 (s, *C*_2_N_3_), 158.75 (s, *C*_2_N_3_), 150.12 (s, aryl *C*H), 146.22 (s, aryl *C*H), 144.79 (s, aryl *C*H), 142.34 (s, aryl *C*H), 140.62 (s, aryl *C*H), 140.02 (s, aryl *C*H), 138.05 (s, aryl *C*H), 137.45 (s, aryl *C*H), –6.67 (s, Al(*C*H_3_)_3_), –8.77 (s, Al(*C*H_3_)_2_). Anal. Calcd for C_23_H_41_Al_4_N_5_(%): C, 55.74; H, 8.34; N, 14.14. Found: C, 55.62; H, 7.16; N, 15.74.

#### Preparation of (trimethylagallium)(3,5-di-2-pyridyl-1,2,4-triazolato)dimethylgallium (½toluene) (2)

In a manner similar to the synthesis of **1**, 3,5-di-2-pyridyl-1,2,4-triazole (0.300 g, 1.340 mmol) was treated with trimethylgallium (0.308 g, 2.680 mmol) in toluene (40 mL). Complex **2** (0.340 g, 58%) was isolated as colorless crystals. Mp 124°C dec.; IR (Nujol, cm^–1^) 3045 (m), 2940 (s), 1601 (s), 1481 (m), 1429 (s), 1266 (s); ^1^H NMR (C_6_D_6_, 18°C, δ) 8.27 (m, 2H, aryl C*H*), 7.62 (m, 2H, aryl C*H*), 7.01 (m, 2.5H, CH_3_C_6_ *H*_5_), 6.88 (m, 2H, aryl C*H*), 6.35 (m, 2H, aryl C*H*), 2.07 (s, 1.5H, C*H*_3_C_6_H_5_), 0.51 (s, 9H, Ga(C*H*_3_)_3_), –0.10 (s, 6H, Ga(C*H*_3_)_2_); ^13^C NMR (C_6_D_6_, 18°C, δ) 156.97 (s, *C*_2_N_3_), 147.42 (s, aryl *C*H), 145.11 (s, aryl *C*H), 138.88 (s, CH_3_ *C*_6_H_5_), 129.27 (s, CH_3_ *C*_6_H_5_), 128.502 (s, CH_3_ *C*_6_H_5_), 125.90 (s, CH_3_ *C*_6_H_5_), 124.66 (s, aryl *C*H), 122.20 (s, aryl *C*H), 21.20 (s, *C*H_3_C_6_H_5_), –3.31 (s, Ga(*C*H_3_)_3_), –6.81 (s, Ga(*C*H_3_)_2_). Anal. Calcd for C_20.5_H_27_Ga_2_N_5_(%): C, 50.99; H, 5.64; N, 14.50. Found: C, 50.92; H, 5.66; N, 14.62.

#### Preparation of (µ-3,5-di-2-pyridyl-1,2,4-triazolato)(µ-methoxy)(tetramethyl)dialuminum (3)

A toluene solution of **1** (0.300 g in 40 mL) was exposed to air undisturbed in a Schlenk flask. After several hours 3 (0.151 g, 68%) precipitated as colorless rod-shaped crystals. Mp 243°C; IR (Nujol, cm^–1^) 3052 (s), 2925 (m), 1590 (w), 1418 (m), 1266 (s), 1105 (m); ^1^H NMR (C_6_D_6_, 18°C, δ) 8.14 (m, 2H, aryl C*H*), 7.81 (m, 2H, aryl C*H*), 6.85 (m, 2H, aryl C*H*), 6.44 (m, 2H, aryl C*H*), 3.66 (s, 3H, OC*H*_3_), –0.18 (s, 12H, Al(C*H*_3_)_2_); ^13^C NMR (C_6_D_6_, 18°C, δ) 162.76 (s, *C*_2_N_3_), 148.33 (s, aryl *C*H), 137.60 (s, aryl *C*H), 125.17 (s, aryl *C*H), 120.06 (s, aryl *C*H), 48.24 (s, O*C*H_3_), –10.25 (s, Al(*C*H_3_)_2_). Anal. Calcd for C_17_H_23_Al_2_N_5_O(%): C, 55.58; H, 6.31; N, 19.06. Found: C, 55.31; H, 6.48; N, 18.86.

#### Preparation of (µ-3,5-di-2-pyridyl-1,2,4-triazolato)(µ-methoxy)(tetramethyl)digallium (4)

In a fashion similar to the synthesis of **3**, **2** (0.300 g in 40 mL) was exposed to air while undisturbed. Complex **4** (0.177 g, 61%) precipitated as colorless rod-shaped crystals. Mp 262°C; IR (Nujol, cm^–1^) 3048 (m), 2920 (s), 1423 (w), 1263 (s), 1095 (s); ^1^H NMR (C_6_D_6_, 18°C, δ) 8.12 (m, 2H, aryl C*H*), 8.07 (m, 2H, aryl C*H*), 6.97 (m, 2H, aryl C*H*), 6.49 (m, 2H, aryl C*H*), 3.58 (s, 3H, OC*H*_3_), 0.22 (s, 12H, Ga(C*H*_3_)_2_); ^13^C NMR (C_6_D_6_, 18°C, δ) 161.43 (s, *C*_2_N_3_), 148.27 (s, aryl *C*H), 137.11 (s, aryl *C*H), 124.46 (s, aryl *C*H), 120.78 (s, aryl *C*H), 49.56 (s, O*C*H_3_), –6.55 (s, Ga(*C*H_3_)_2_). Anal. Calcd for C_17_H_23_Ga_2_N_5_O(%): C, 45.09; H, 5.12; N, 15.47. Found: C, 44.85; H, 5.01; N, 15.22.

## Associated Content

### Crystallographic Data

Crystallographic data (excluding structure factors) for the structures reported in this paper have been deposited with the Cambridge Crystallographic Data Centre as supplementary publication nos. CCDC 989107–989110. Copies of the data can be obtained free of charge on application to CCDC, 12 Union Road, Cambridge CB2 1EZ, UK (fax: (+44) 1223-336-033; e-mail: deposit@ccdc.cam.ac.uk).
